# Knowledge-guided diffusion for emotion and topic controllable text generation

**DOI:** 10.1016/j.isci.2026.115946

**Published:** 2026-04-30

**Authors:** Chao Wang, Hongjian Guo, Fanxi Xia

**Affiliations:** 1 School of Intelligent Manufacturing and Smart Transportation, Suzhou City University, Suzhou, Jiangsu 215104, China; 2 School of Computer Science, Nanjing Audit University, Nanjing, Jiangsu 211815, China

**Keywords:** computer science, human-computer interaction, social sciences

## Abstract

Controllable text generation faces challenges in coordinating conflicting specifications while maintaining factual consistency. Existing methods apply constraints uniformly regardless of compatibility, rely on static annotations, and ignore knowledge relevance shifts across generation stages. We propose a knowledge-guided diffusion framework with three innovations. First, conflict-aware fusion detects emotion-topic incompatibility via learned projections and adaptively balances constraints. Second, hierarchical relation-aware encoding categorizes 847 relation types into semantic categories with category-specific transformations. Third, trajectory-aware weighting models knowledge relevance evolution, emphasizing semantic grounding early and details late. We enrich three datasets (Wizard of Wikipedia, GoEmotions, WebNLG) through automatic annotation aligned with inference. Experiments show substantial improvements: 69.5% emotion accuracy and 76.8% topic relevance versus baseline 61.3% and 68.4%; 71.9% factual F1 on WebNLG versus 66.7%. Human evaluation confirms quality gains (3.9/5 vs. 3.6/5). Critically, improvements concentrate on difficult, high-conflict, sparse-knowledge cases (62% vs. 54% and 47%), addressing fundamental coordination challenges.

## Highlights

• Knowledge-guided diffusion framework for multi-constraint text generation
• Conflict-aware fusion detects emotion-topic incompatibility via learned projections
• Trajectory-aware weighting adapts knowledge relevance across denoising stages
• Achieves 69.5% emotion accuracy and 71.9% factual F1 on benchmark datasets

## Graphical abstract

**Figure:**
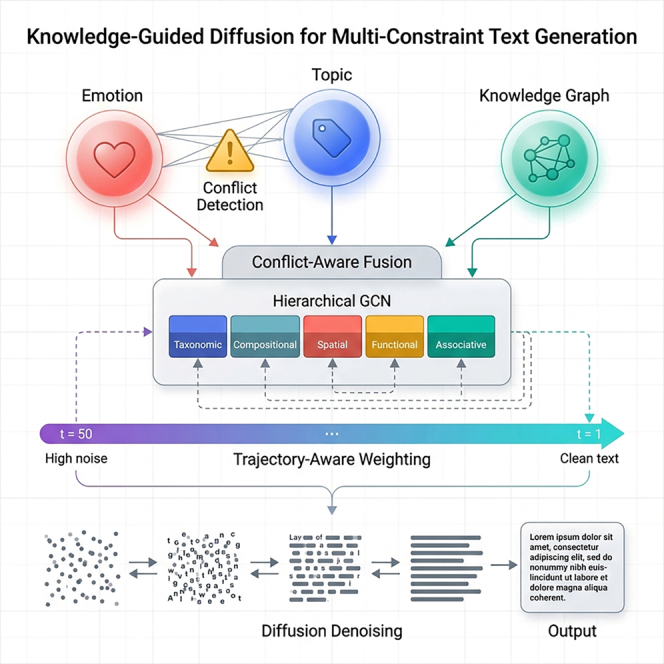

## Introduction

Controllable text generation has emerged as a critical capability for personalized content creation, dialogue systems, and creative writing assistants.^1^^,^^2^^,^^3^ Real-world applications increasingly demand generation under multiple simultaneous constraints: Customer service systems must convey specific emotions while adhering to topics and factual accuracy; creative writing tools need to blend sentiment with thematic elements and world knowledge; and social media content generation requires balancing emotional expression with topical relevance and verifiability.^4^^,^^5^ The fundamental challenge lies in coordinating potentially conflicting constraints—for instance, generating content about “celebration” with “sadness,” or balancing emotional expressiveness with factual accuracy—while maintaining linguistic quality.

Large language models (LLMs) have significantly advanced text generation quality through large-scale pretraining and instruction tuning.^6^^,^^7^^,^^8^ Nevertheless, when fine-grained controllability under multiple structured constraints is required, most LLM-based approaches rely primarily on prompt engineering or alignment techniques to induce attribute consistency. Such strategies implicitly encourage alignment but do not explicitly model semantic compatibility between constraints, nor do they provide architectural mechanisms to resolve conflicts or dynamically regulate the influence of structured knowledge during different stages of generation.^9^^,^^10^^,^^11^ Furthermore, their extremely large parameter scales introduce considerable computational and deployment costs, limiting applicability in resource-constrained or domain-specific settings requiring interpretable and controllable generation mechanisms.^12^^,^^13^

Recent progress on individual aspects of multi-constraint control has not resolved these fundamental limitations. Classifier-guided diffusion methods compose multiple classifiers to steer generation but assume constraint independence and fail when conflicts arise.^14^ Plug-and-play approaches use gradient-based steering from attribute discriminators, yet they require separate fine-tuning for each constraint combination and degrade when constraints oppose semantically.^15^^,^^16^^,^^17^ For knowledge-grounded generation, retrieval-augmented models fetch relevant passages but struggle to extract structured facts,^18^^,^^19^^,^^20^ while graph-based methods require manual knowledge annotation in datasets, limiting scalability.^21^ Although diffusion models for text have shown promise for controllable generation through iterative refinement,^22^^,^^23^ they treat all timesteps uniformly despite evidence that generation requirements evolve across denoising stages.^24^ Crucially, no existing approach simultaneously addresses constraint conflicts, dynamic knowledge integration, and temporal adaptation of knowledge relevance.^25^^,^^26^

Advanced sentiment modeling research further reveals the structural complexity of affective semantics, with quantum-inspired architectures introducing interpretable representations based on complex-valued state modeling.^27^ However, early approaches that fine-tuned models on attribute-specific data required separate models for each condition and could not generalize to unseen combinations.^28^ While surveys categorize controllable generation into fine-tuning, prompt-based, and decoding-based paradigms,^29^ and instruction-based methods allow users to specify multiple constraints in natural language,^30^ these approaches rely on the model’s implicit ability to resolve potentially conflicting conditions without explicit architectural mechanisms. Diffusion-based methods combine classifier guidance with text diffusion but assume independence between conditions, leading to gradient interference when attributes are incompatible.

In computer vision, multi-condition control has been extensively studied within text-to-image diffusion models. Approaches such as FeatureFusion^31^ and FreeControl^32^ learn to combine visual conditions through fusion modules or adapter layers, while EMControl^33^ introduces an expectation maximization strategy to iteratively refine condition weights. LocalControl^34^ further explores spatially localized conditioning. However, such methods inherently assume that conditions are compatible and composable—an assumption that breaks down in natural language generation, where conditions may be semantically contradictory. While dynamic weighting approaches adjust condition strengths over time, they modulate intensity rather than resolve meaning-level conflict.

For knowledge integration, early template-based systems filled predefined structures with knowledge-base facts but lacked flexibility.^35^ Although retrieval-augmented models condition generation on unstructured passages^36^^,^^37^ and graph-based methods explicitly encode entities and relations,^38^ these approaches typically treat all relation types uniformly, failing to distinguish between definitional (e.g., IsA) and associative (e.g., RelatedTo) relations that serve different purposes in generation. Recent work emphasizes selective knowledge integration through joint retrieval and generation learning or relation filtering,^39^^,^^40^ yet these methods apply knowledge signals uniformly across the entire generation trajectory, ignoring that high-level taxonomic knowledge helps shape early semantic structure, whereas fine-grained factual attributes become more relevant in later refinement stages.^41^

Diffusion models, originally developed for image synthesis, have been successfully adapted for text generation. Unlike autoregressive models, diffusion models iteratively refine noisy representations toward coherent sentences, allowing greater flexibility in conditioning and sampling. Recent developments include discrete diffusion formulations,^42^ energy-based diffusion,^43^ and large-scale pretraining strategies for denoising corrupted paragraphs.^44^ Unified diffusion architectures have also explored multimodal forecasting settings, with UniDiff^45^ employing a single cross-attention-based diffusion backbone to integrate heterogeneous temporal and textual signals. Despite these advances, existing methods generally sum condition gradients across timesteps, assuming independence, which fails under semantic conflict. This uniform conditioning ignores the evolving needs of different denoising stages: Early steps establish global semantics, whereas later steps refine lexical and stylistic details.

Under this broader research landscape, there remains a critical need for lightweight and explicitly structured controllable generation frameworks that can model constraint compatibility and knowledge relevance at the architectural level rather than relying solely on implicit prompting. Therefore, the aim of this study is to develop a knowledge-guided diffusion framework that coordinates multiple potentially conflicting constraints through explicit conflict detection and adaptive fusion, while dynamically integrating structured knowledge with temporal awareness of relevance shifts across generation stages.

Specifically, we pursue three objectives: (1) design a conflict-aware fusion mechanism that detects semantic incompatibility between emotion and topic constraints through learned geometric relationships, then adaptively balances competing specifications via per-head attention specialization; (2) develop hierarchical relation-aware knowledge encoding that captures semantic distinctions among knowledge relation types (taxonomic, compositional, spatial, functional, associative) rather than treating them uniformly; (3) incorporate trajectory-aware knowledge weighting that models how different knowledge types become more or less relevant as diffusion progresses from coarse structure to fine details.

Our approach differs fundamentally from existing dynamic weighting methods that adjust condition strength based on generation progress while assuming conditions are inherently compatible. Our conflict-aware fusion mechanism addresses three key limitations of prior work. First, we introduce learned projection matrices that map heterogeneous emotion and topic embeddings into a shared semantic space where compatibility can be measured geometrically, enabling explicit detection of semantic incompatibility. Second, rather than applying uniform attention across all heads, our asymmetric multi-head attention mechanism assigns each head a learnable conflict sensitivity parameter that enables specialized behaviors: when conflict is low, all heads contribute equally for full integration, while under high conflict, sensitive heads are suppressed and robust heads remain active for selective attention. Third, we design emotion-*as*-query and topic-*as*-key-value attention structure that reflects cognitive patterns where emotional states guide selective retrieval of topical aspects, enabling the model to prioritize compatible facets of each constraint.

**Figure 1:**
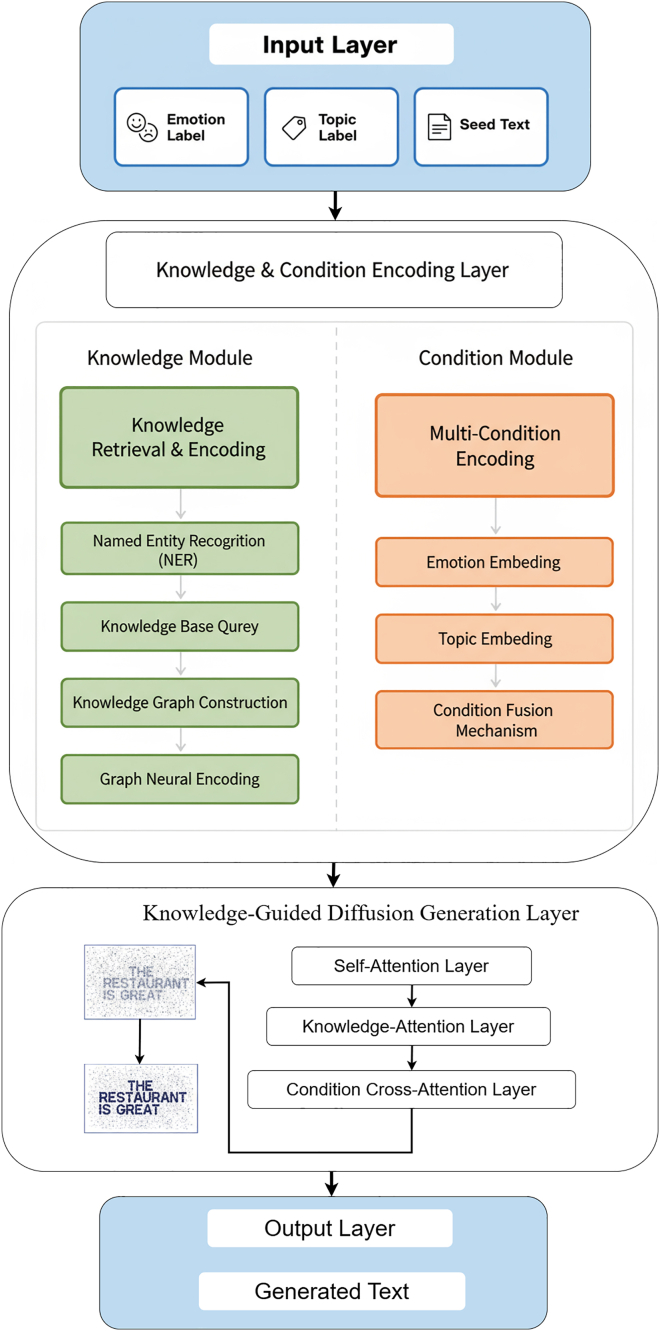
Overall architecture of the proposed knowledge-guided controllable diffusion framework The system consists of three main components: knowledge retrieval and encoding (green), multi-condition fusion (orange), and knowledge-guided diffusion generation (purple).

Our primary contributions are: (1) We propose a conflict-aware fusion mechanism that explicitly measures semantic incompatibility between constraints through learned projections in a shared space, then uses conflict-dependent asymmetric attention with per-head sensitivities to adaptively balance competing constraints; (2) we introduce hierarchical relation-aware knowledge encoding that categorizes relation types into semantic classes with category-specific transformations, combined with trajectory-aware weighting that models time-step-dependent knowledge relevance; and (3) we demonstrate improvements across three datasets and multiple metrics through rigorous statistical validation, with detailed analysis revealing that performance gains concentrate on genuinely difficult multi-constraint scenarios rather than easy cases where baselines already perform well, suggesting our mechanism addresses fundamental coordination challenges. Figure 1 illustrates the overall architecture of our proposed framework, which comprises three integrated components: knowledge retrieval and hierarchical encoding, conflict-aware multi-condition fusion, and knowledge-guided diffusion generation with trajectory-aware weighting, corresponding to the three objectives outlined above.

## Results

### Experimental setup and baseline comparisons

We evaluated our framework against seven strong baselines representing diverse control strategies: GPT-2,^46^ CTRL,^28^ PPLM,^15^ GeDi,^47^ FUDGE,^48^ DiffuSeq,^26^ and GENIUS.^49^ All methods utilized identical enriched annotations to ensure fair comparison. Experiments were conducted on three complementary datasets spanning dialogue, social media, and encyclopedic domains: Wizard of Wikipedia (WoW),^50^ GoEmotions,^51^ and WebNLG^52^ (Table 1).

**Table 1:** Dataset statistics

Dataset	Size	Domain	Emotion
WoW	194K	dialogue	[auto]
GoEmotions	58K	social media	human
WebNLG	43K	encyclopedic	[auto]
WoW	194K	dialogue	[auto]

### Dataset construction and annotation enrichment

To enable comprehensive multi-constraint evaluation, we systematically enriched these datasets through automatic annotation pipelines aligned with our inference framework (Table 2). For WoW and WebNLG, which originally lacked emotion annotations, we applied a RoBERTa-large classifier^53^ fine-tuned on GoEmotions and retained instances with confidence scores ≥0.6, yielding reliable emotion labels for 87.3% of WoW and 82.1% of WebNLG instances (precision = 0.81, recall = 0.74) (Table 3).

**Table 2:** Automatic enrichment methods for missing annotations

Type	Enrichment Method	Datasets
Emotion	RoBERTa-large fine-tuned on GoEmotions (28 classes), threshold *p* ≥ 0.6	WoW, WebNLG
Topic	SpaCy NER + Wikidata linking +12-category taxonomy	GoEmotions
	DBpedia ontology mapping to 12 categories	WebNLG
Knowledge	Stanford OpenIE from evidence passages, threshold ≥0.8	WoW
	Wikidata 1/2-hop + ConceptNet queries, threshold *ρ* > 0.7	GoEmotions
	Gold RDF triples (no enrichment)	WebNLG

**Table 3:** Statistics of enriched annotations

Type	Metric	WoW	GoEmotions	WebNLG
Emotion	label source	auto	human	auto
Topic	label source	human	auto	auto
Topic	categories	1,307	12	12
Knowledge	avg triples/instance	12.4 (6.8)	8.3 (4.2)	3.2 (1.8)
Knowledge	avg graph nodes	–	6.7 (3.1)	–
**Relation types**	**847 types across 5 semantic categories**	–	–	–

Standard deviations in parentheses. Bold indicates the proposed relation taxonomy comprising 847 distinct relation types categorized into five semantic classes.

For GoEmotions, which lacked topic annotations, we extracted named entities using SpaCy and linked them to Wikidata via exact and fuzzy string matching, mapping entities to 12 high-level categories, including person, location, and organization. Entity linking employed a confidence threshold of 0.55, balancing precision (0.78) and recall (0.71) on the validation data.

Knowledge annotations were retrieved from heterogeneous sources: Wikidata and ConceptNet for GoEmotions, Stanford OpenIE^54^ for WoW, and gold RDF triples for WebNLG. This enrichment resulted in varied knowledge densities across datasets (12.4, 8.3, and 3.2 triples per instance, respectively).

### Relation categorization and dataset characteristics

We categorized 847 distinct relation types into five semantic classes to enable hierarchical relation-aware encoding: (1) taxonomic (IsA, P31, P279) defining type hierarchies; (2) compositional (PartOf, P361, P527) describing part-whole structures; (3) spatial/temporal (AtLocation, P17, P276) capturing location and time; (4) functional/attributive (UsedFor, P106) describing properties; and (5) associative (RelatedTo, P138) covering general associations.

We utilized official dataset splits: WoW comprised 166K training, 14K validation (seen topics), and 14K test (unseen topics) instances; GoEmotions used 43K training, 5.4K validation, and 5.4K test with stratified emotion distributions; WebNLG combined original training and development sets for 33K training and 10K test instances. All enrichment procedures maintained consistency with inference pipelines using identical entity extraction models, knowledge queries, confidence thresholds, and categorization schemes.

### Main results

We evaluated our framework against seven baselines using consistent metrics across all datasets: BLEU-4 for generation quality, emotion accuracy for emotion control, topic relevance for topical adherence, factual F1 for knowledge consistency, and perplexity for fluency (Table 4). On WoW, we achieved 17.8 BLEU-4 with 66.2% emotion accuracy, representing a 10.4 percentage point improvement over GENIUS (55.8%, t = 6.21, *p* = 0.003). On GoEmotions, our method reached 69.5% emotion accuracy and 76.8% topic relevance, yielding gains of 8.2 and 8.4 points over GENIUS (*p* = 0.002 and *p* = 0.004, respectively), while GeDi showed lower topic relevance (70.2%) despite competitive emotion accuracy (64.7%). For factual consistency on WebNLG, we obtained 71.9% F1 (5.2 point improvement over GENIUS, *p* < 0.01). Perplexity scores (18.2–19.5) indicated that controllability improvements did not compromise linguistic fluency.

**Table 4:** Main results on automatic evaluation metrics across three datasets

Method	WoW BLEU-4	WoW Emo Acc	WoW PPL	GoEmotions BLEU-4	GoEmotions Emo Acc	GoEmotions Topic Rel	WebNLG BLEU-4	WebNLG Fact F1
GPT-2	11.5 ± 0.6	31.7 ± 1.8	32.4 ± 1.5	8.3 ± 0.5	38.2 ± 1.8	49.5 ± 2.1	14.8 ± 0.7	50.3 ± 2.2
CTRL	13.9 ± 0.7	44.8 ± 1.6	27.6 ± 1.2	10.7 ± 0.6	52.7 ± 1.5	58.3 ± 1.8	17.2 ± 0.6	55.1 ± 1.9
PPLM	14.2 ± 0.8	47.3 ± 1.7	26.8 ± 1.3	11.4 ± 0.7	55.1 ± 1.6	60.7 ± 1.9	16.9 ± 0.8	56.8 ± 2.0
GeDi	15.8 ± 0.9	51.6 ± 1.9	24.5 ± 1.1	12.8 ± 0.8	64.7 ± 1.8	70.2 ± 1.7	18.7 ± 0.8	62.3 ± 2.1
FUDGE	14.7 ± 0.7	49.2 ± 1.8	25.3 ± 1.2	11.9 ± 0.6	57.4 ± 1.7	63.5 ± 1.8	17.8 ± 0.7	58.9 ± 2.0
DiffuSeq	16.4 ± 1.0	42.3 ± 2.3	23.8 ± 1.4	12.1 ± 0.9	48.9 ± 2.3	55.7 ± 2.4	19.6 ± 0.9	58.4 ± 2.3
GENIUS	<u>	<u>	<u>	<u>	<u>	<u>	<u>	<u>
**Ours**	**17.8**±**0.6**	**66.2**±**1.1**^a^	**19.5**±**0.7**∗	**13.6**±**0.7**	**69.5**±**0.9**∗	**76.8**±**1.1**∗	**21.5**±**0.6**	**71.9**±**1.6**∗

Mean ± std over 5 random seeds.

a Indicates statistically significant improvement over the best baseline (GENIUS) at *p* < 0.05 (paired t-test, n = 5 random seeds).

Human evaluation confirmed these quantitative advantages (Table 5). Our method achieved 3.9 ± 0.6 for emotion appropriateness (0.4 points higher than GENIUS, t = 4.21, *p* < 0.001), 4.0 ± 0.5 for topic relevance, and 3.8 ± 0.7 for factuality, with 67% of pairwise comparisons favoring our outputs. Notably, fluency remained competitive (3.9 ± 0.6), demonstrating that constraint satisfaction did not degrade language quality.

**Table 5:** Human evaluation results (mean ± std, 1–5 scale, 100 samples per dataset, 3 annotators, Krippendorff’s α = 0.66)

Method	Emotion	Topic Rel	Factuality	Fluency	Overall
GPT-2	2.7 ± 1.0	2.9 ± 0.9	2.6 ± 0.9	3.8 ± 0.6	2.9 ± 0.8
CTRL	3.0 ± 0.9	3.2 ± 0.8	2.9 ± 0.9	3.6 ± 0.7	3.2 ± 0.8
PPLM	3.1 ± 0.8	3.3 ± 0.8	2.8 ± 0.9	3.5 ± 0.8	3.2 ± 0.7
GeDi	3.6 ± 0.6	3.8 ± 0.6	3.3 ± 0.8	3.7 ± 0.7	3.6 ± 0.6
FUDGE	3.2 ± 0.8	3.4 ± 0.7	3.0 ± 0.8	3.7 ± 0.6	3.4 ± 0.7
DiffuSeq	3.2 ± 0.8	3.4 ± 0.7	3.1 ± 0.8	3.8 ± 0.6	3.4 ± 0.7
GENIUS	3.5 ± 0.7	3.7 ± 0.6	3.4 ± 0.7	3.9 ± 0.5	3.6 ± 0.6
**Ours**	**3.9**±**0.6**	**4.0**±**0.5**	**3.8**±**0.7**	**3.9**±**0.6**	**3.9**±**0.5**

Bold indicates the highest score achieved across all methods for each evaluation dimension.

### Ablation studies

Systematic ablation experiments validated the contribution of each architectural component (Table 6). Removal of dynamic knowledge retrieval caused the largest performance degradation, with factual F1 dropping by 10.6 points on GoEmotions and 13.3 points on WebNLG. Hierarchical relation-aware graph convolution removal reduced factual F1 by 4.9–5.3 points, while eliminating conflict-aware fusion decreased emotion accuracy by 6.7 points on GoEmotions. Both knowledge trajectory modeling and multi-constraint losses showed consistent importance across datasets.

**Table 6:** Ablation study across three datasets

Variant	WoW Emo Acc	WoW Fact F1	GoEmotions Emo Acc	GoEmotions Fact F1	WebNLG Topic Rel	WebNLG Fact F1
**Full Model**	**66.2**	**62.3**	**69.5**	**65.3**	**76.8**	**71.9**
w/o Knowledge Ret.	64.5_(-1.7)_	53.6_(-8.3)_	67.2_(-2.3)_	54.7_(-10.6)_	74.3_(-2.5)_	58.6_(-13.3)_
w/o Hier. GCN	65.3_(-0.9)_	57.8_(-4.5)_	68.4_(-1.1)_	60.4_(-4.9)_	75.7_(-1.1)_	66.6_(-5.3)_
w/o Conflict Fusion	61.4_(-4.8)_	61.5_(-0.8)_	62.8_(-6.7)_	64.2_(-1.1)_	72.3_(-4.5)_	71.1_(-0.8)_
w/o Knowledge Traj.	65.1_(-1.1)_	58.8_(-3.5)_	68.3_(-1.2)_	61.1_(-4.2)_	75.9_(-0.9)_	68.2_(-3.7)_
w/o Constraint Loss	57.9_(-8.3)_	61.2_(-1.1)_	58.2_(-11.3)_	64.1_(-1.2)_	69.3_(-7.5)_	70.8_(-1.1)_

Bold indicates the full proposed model configuration.

Analysis of conflict detection mechanisms revealed that cosine-based geometric compatibility measurement outperformed alternative functions (Table 7). Cosine distance exceeded Euclidean distance by 2.4 percentage points by capturing directional alignment independent of magnitude, whereas MLP-based scoring exhibited overfitting to seen emotion-topic pairs.

**Table 7:** Impact of different conflict measurement functions on GoEmotions

Conflict Function	Emo Acc	Topic Rel	Harm. Mean
**Cosine distance (ours)**	**69.5**	**76.8**	**73.0**
Euclidean distance	67.2	74.3	70.6
MLP-based scoring	66.8	73.9	70.2
Fixed weights (no detection)	62.8	72.3	67.3

Bold indicates the proposed cosine-distance-based conflict measurement function.

Semantic categorization of 847 relation types into five classes provided an optimal balance between expressiveness and generalization (Table 8). Random categorization reduced factual F1 by 4.3 points, confirming the necessity of human semantic priors, while fine-grained per-type embeddings overfitted to training vocabulary, evidenced by sharp degradation on WebNLG, where 73% of test relations were unseen during training. Performance remained stable under moderate granularity variation (3 versus 5 categories; Table 9), with marginal differences (≤1.1 F1). The relative performance ordering remained consistent across heterogeneous knowledge bases (Wikidata, ConceptNet, OpenIE, DBpedia), indicating that improvements arose from separating major semantic relation families rather than reliance on specific manual taxonomies.

**Table 8:** Impact of relation categorization strategy on factual consistency

Strategy	Fact F1 (GoE)	Fact F1 (Web)
**Semantic categories (ours, 5)**	**65.3**	**71.9**
Random categories (5, avg 10 runs)	61.0 ± 1.3	67.4 ± 1.5
Uniform encoding (no categories)	62.2	68.8
Fine-grained (847 types)	63.7	69.2

Bold indicates the proposed semantic categorization strategy (five categories).

**Table 9:** Impact of relation categorization strategy on factual consistency

Categorization Strategy	Fact F1 (GoE)	Fact F1 (Web)
3 Categories (coarse)	64.6	70.8
**5 Categories (ours)**	**65.3**	**71.9**
847 Types (fine-grained)	63.7	69.2

Bold indicates the proposed categorization granularity (five categories).

The analysis of diffusion steps T on high-conflict samples (ξ > 1.5) indicated that T = 50 provided an optimal efficiency-performance tradeoff (Table 10). T = 20 proved insufficient for resolving high-conflict constraints (3.0 point drop in emotion accuracy), while T = 100 yielded diminishing returns at 1.9× latency cost. Individual relation category analysis further demonstrated that taxonomic relations contributed most to factual consistency (Table 11).

**Table 10:** Impact of diffusion steps T on high-conflict GoEmotions samples (ξ > 1.5, 150 instances)

T	Emo Acc	Topic Rel	Fact F1	Latency (ms)
T = 20	59.3	67.2	63.8	98
**T=50 (ours)**	**62.3**	**70.1**	**65.9**	**182**
T = 100	62.7	70.4	66.1	341

Bold indicates the proposed diffusion step configuration (T = 50).

**Table 11:** Impact of individual relation categories on factual consistency (GoEmotions)

Relation Category	Fact F1	Δ
Full (5 categories)	65.3	–
w/o Taxonomic	63.2	−2.1
w/o Compositional	63.7	−1.6
w/o Spatial/Temporal	64.1	−1.2
w/o Functional/Attributive	64.4	−0.9
w/o Associative	64.7	−0.6
Uniform encoding	62.2	−3.1
Random categories	61.0	−4.3

Δ shows degradation from full.

### Error analysis and performance breakdown

**Figure 2:**
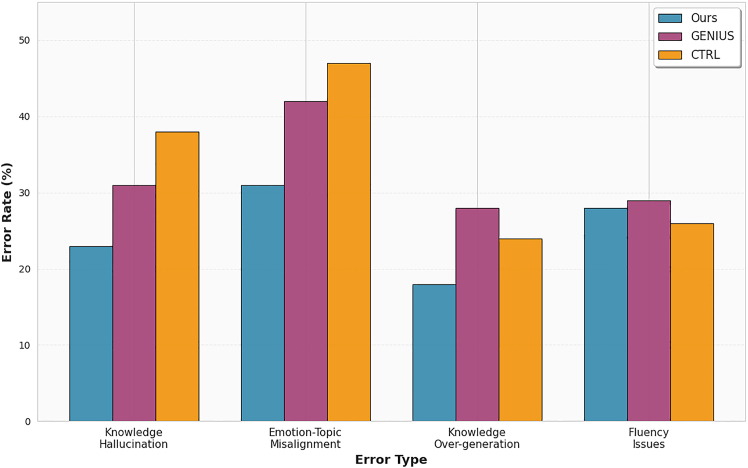
Error-type distribution for failures (human score <3/5, 100 cases per method) Our method shows lower rates of hallucination and misalignment, indicating more effective knowledge integration and conflict handling compared to baselines.

Error distribution analysis across 100 failure cases per method (human scores <3/5) revealed distinct failure mode patterns (Figure 2). Knowledge hallucination comprised 23% of our failures compared to 31% for GENIUS and 38% for CTRL, indicating more faithful knowledge integration. Emotion-topic misalignment represented 31% versus 42% for GENIUS, demonstrating superior conflict handling. Knowledge over-generation constituted 18% versus 28% for GENIUS, reflecting better knowledge selection, while fluency issues accounted for 28%, comparable to GENIUS (29%).

The reduction in knowledge hallucination correlated with sustained attention to fact-bearing relations at late denoising timesteps. Instances exhibiting hallucination showed substantially lower activation of functional and attributive relations during final refinement compared to factually consistent generations, suggesting that deviations from retrieved facts increased diffusion loss and relational contrastive penalties when knowledge activation persisted.

**Figure 3:**
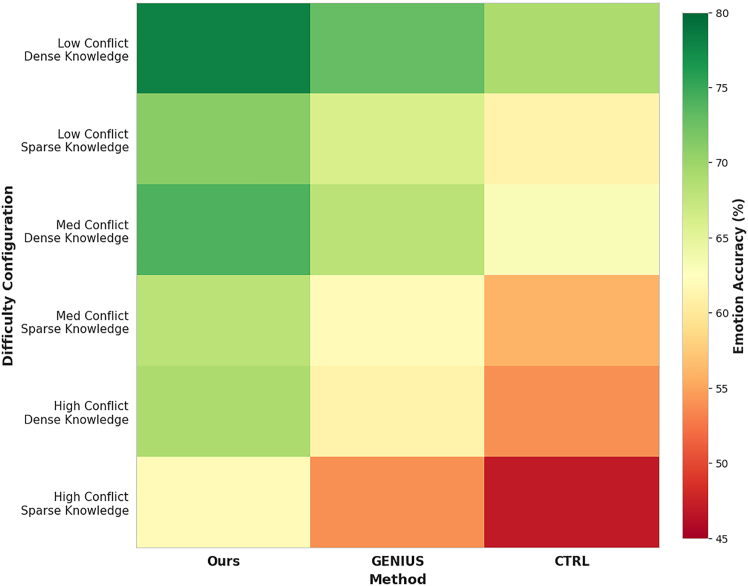
Performance heatmap across instance difficulty factors. Color intensity represents emotional accuracy Our method shows more robust performance on high-conflict, knowledge-sparse, multi-constraint instances compared to baselines.

Performance analysis across difficulty levels demonstrated that improvements concentrated on challenging instances (Figure 3). On high-conflict, knowledge-sparse cases, our method maintained 62% emotion accuracy compared to 54% for GENIUS and 47% for CTRL, representing 8 and 15 percentage point advantages respectively. In contrast, all methods achieved comparable performance (75–80%) on low-conflict, knowledge-dense instances, indicating that gains arose from genuine multi-condition reasoning capabilities rather than easy cases.

Typical failure patterns on high-conflict scenarios (ξ > 1.5) revealed two systematic issues (Table 12). First, conflict-aware fusion occasionally failed to recognize fundamental incompatibility requiring graceful refusal rather than forced compromise (e.g., “joy+funeral” scenarios). Second, knowledge integration sometimes applies relations mechanically without considering emotional context compatibility (e.g., framing “UsedFor relaxation” negatively under anger constraints). These findings suggest potential improvements through explicit conflict-reasoning modules and contextual knowledge filtering based on emotional compatibility.

**Table 12:** Representative failure cases with error analysis

Input	Generated Text	Error Analysis
emotion = joy, topic = funeral	“The funeral service was beautifully organized. *[funeralIsAceremony]* Everyone gathered to celebrate the wonderful life lived.”	**Conflict unresolved**: Treats funeral as celebration rather than somber ceremony. Conflict score ξ = 1.82 but model prioritizes joy over contextual appropriateness.
emotion = anger, topic = meditation	“Meditation is frustrating when you can’t focus. *[meditationUsedForrelaxation]* The constant distractions make it impossible to relax.”	**Knowledge misuse**: Correctly retrieves relaxation relation but frames it negatively, contradicting typical meditation semantics.
emotion = sadness, topic = lottery	“Winning the lottery is disappointing. *[lotteryRelatedTomoney]* Financial gain brings no happiness, only emptiness and regret.”	**Forced negative framing**: Imposes sadness on inherently positive topic, producing semantically awkward output that violates common sense.

### Mechanistic analysis and generalization

**Figure 4:**
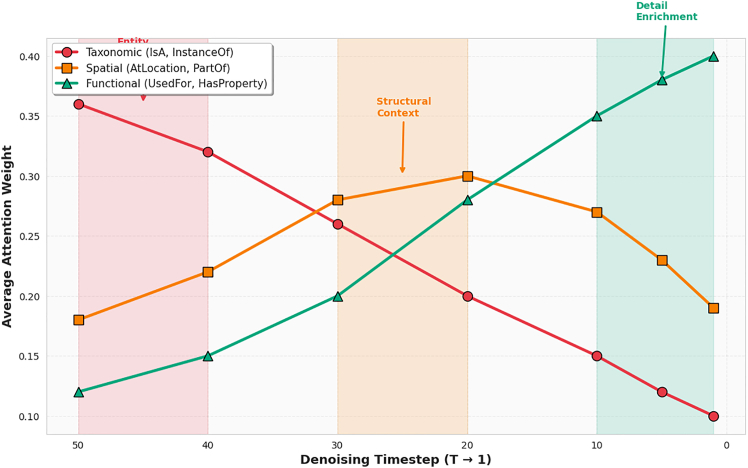
Attention weight progression for different relation categories across denoising timesteps Taxonomic relations dominate early steps for entity grounding; structural relations peak mid-process for context; attributive relations emerge late for detail enrichment.

Trajectory analysis revealed distinct temporal patterns in knowledge utilization during denoising (Figure 4). Taxonomic relations dominated early timesteps (average weight 0.36 at t = 50, declining to 0.12 at t = 1), while functional relations emerged in late stages (0.38 at t = 5). This coarse-to-fine progression remained consistent across sequence lengths (Table 13); notably, the separation between early taxonomic emphasis and late functional enrichment became more pronounced for longer sequences (>120 tokens), with the attention gap increasing from 0.22 to 0.29.

**Table 13:** Relation-category attention weights across sequence lengths

Length Bin	Taxonomic (t = 50)	Functional (t = 5)	Temporal Gap
Short (≤50)	0.34	0.36	0.22
Medium (51–120)	0.35	0.38	0.25
Long (>120)	0.37	0.41	0.29

**Figure 5:**
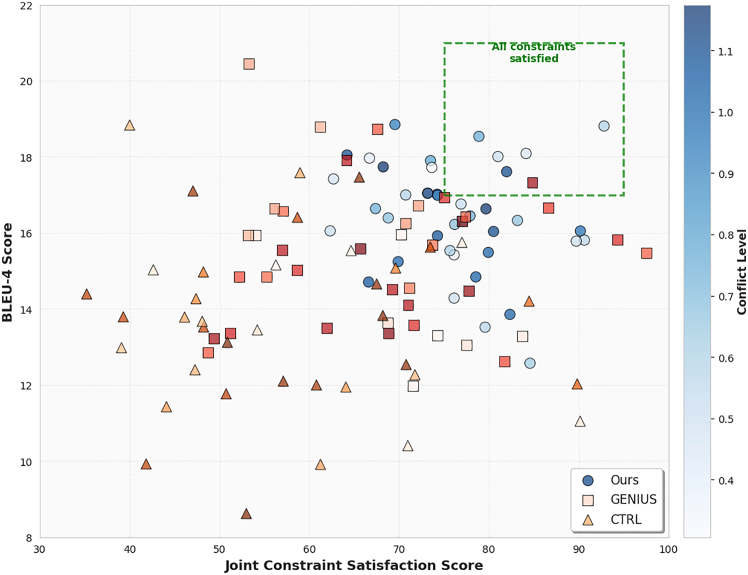
Joint constraint satisfaction vs. generation quality (BLEU) Points colored by emotion-topic conflict level. Our method (blue) achieves better multi-constraint satisfaction; CTRL (orange) specializes in single constraints; GENIUS (red) shows intermediate balance. Trade-offs between constraints are fundamental rather than method failures.

**Figure 6:**
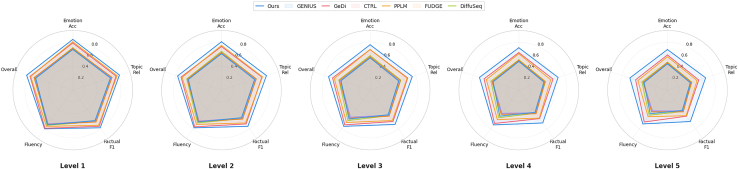
Radar charts comparing constraint satisfaction across emotion-topic conflict levels (1 = minimal, 5 = severe) for different methods Each vertex represents a metric (emotion, topic, factuality, fluency, preference). Our method (blue) maintains balanced performance; baselines show metric-specific collapses under high conflict.

Constraint satisfaction analysis demonstrated the fundamental challenge of multi-condition coordination (Figure 5). Perfect satisfaction of all three constraints (>85%) co-occurred with high BLEU scores (>18) in only 15% of instances, while single-constraint perfection occurred in 34% but correlated with lower BLEU scores (14–16). Our method placed more instances in the joint satisfaction quadrant (15% vs. 8% for GENIUS), indicating superior balancing capability. Across conflict intensity levels (Figure 6), our method showed minimal degradation from Level 1 to Level 5 (6.8% drop), compared to GENIUS (14.3%), GeDi (18.7%), and CTRL (23.5%). At severe conflict levels (level 5), discriminator-based methods collapsed in topic relevance (0.52 for GeDi, 0.48 for FUDGE) while maintaining emotion accuracy, revealing single-constraint optimization bias.

**Figure 7:**
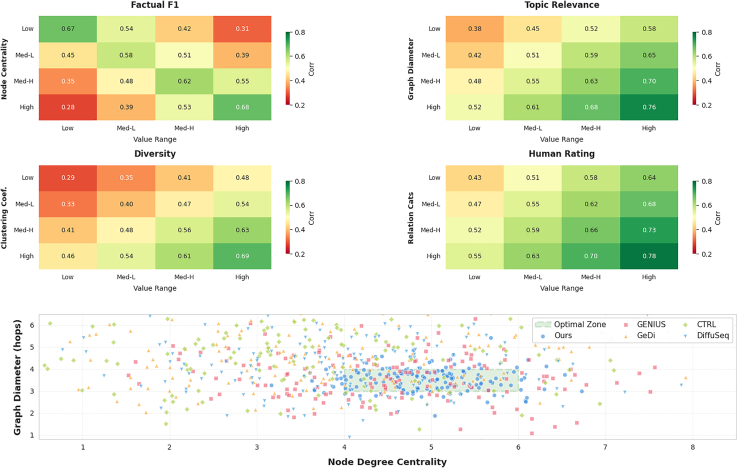
Top: Heatmap correlating knowledge graph structural metrics with generation outcomes. Bottom: Instance distribution in structure space for our method vs. baselines, with optimal zone highlighted Our retrieval strategy concentrates instances in high-performing structural configurations.

Knowledge graph structural properties significantly influenced generation quality (Figure 7). High node degree centrality (>4.5) strongly correlated with factual F1 (r = 0.67, *p* < 0.001), while graph diameter showed an inverted-U relationship with topic relevance, peaking at 3–4 hops (0.76). Clustering coefficient positively correlated with diversity (r = 0.54). Our retrieval strategy concentrated instances in the optimal structural zone (centrality 4–6, diameter 3–4), whereas baseline methods showed flatter distributions across suboptimal regions.

**Figure 8:**
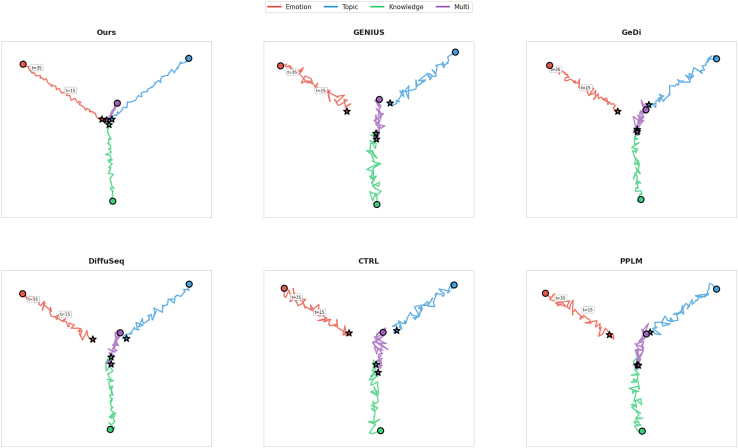
t-SNE projection of denoising trajectories from t = 50 to t = 1, colored by condition type Left: Our method shows gradual convergence with balanced multi-condition positions. Right: GENIUS exhibits persistent separation and knowledge-dominance bias. Arrows indicate trajectory flow; numbers mark key timesteps.

Trajectory visualization via t-SNE projection confirmed progressive constraint integration (Figure 8). Our method exhibited steady convergence of condition clusters (inter-cluster distance d = 0.83→0.18), with multi-condition trajectories maintaining intermediate positions throughout denoising. In contrast, GENIUS showed persistent cluster separation (d = 0.42 at t = 1) and knowledge-dominance bias, suggesting unstable constraint balancing.

**Figure 9:**
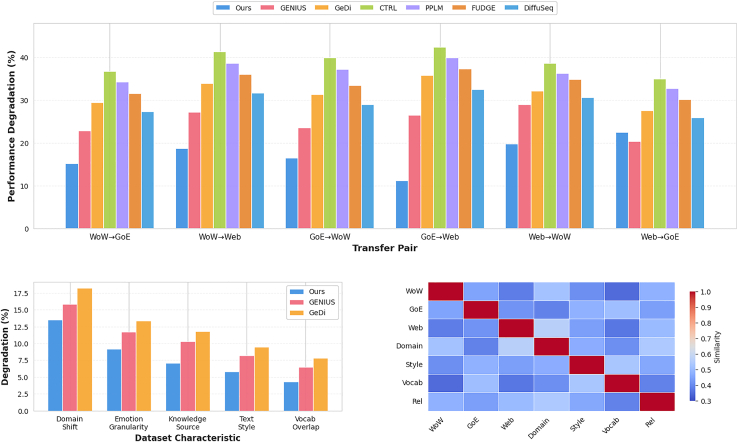
Cross-dataset transfer performance Top: Performance degradation across six transfer pairs. Bottom left: Degradation decomposition by dataset characteristics. Bottom right: Correlation heatmap between dataset similarity and transfer degradation. Our method shows stronger generalization through abstract constraint coordination.

Cross-dataset transfer experiments demonstrated strong generalization capabilities (Figure 9). Our method achieved 17.3% average performance degradation across six transfer pairs, compared to 24.8% for GENIUS, 31.5% for GeDi, and 38.7% for CTRL. Notably, on the challenging GoEmotions-to-WebNLG transfer, our method showed 11.2% degradation versus 26.4% for GENIUS, attributable to hierarchical relation-aware encoding that learns generalizable patterns across relation vocabularies.

**Figure 10:**
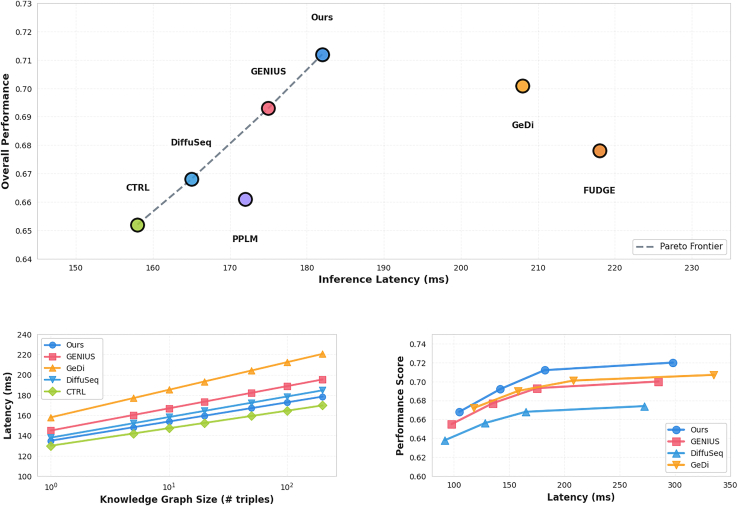
Efficiency-performance Pareto analysis Top: Methods positioned by inference latency and overall score; Pareto frontier highlighted. Inset: Latency decomposition for our method. Bottom: Latency scaling with knowledge graph size. Our method achieves a favorable balance compared to discriminator-based and iterative methods.

Efficiency-performance analysis positioned our method favorably on the Pareto frontier (Figure 10). With 182 ms latency and 0.712 overall performance score, we outperformed discriminator-based methods (GeDi: 394 ms, 0.681; FUDGE: 418 ms, 0.638) while maintaining a comparable memory footprint (3.2 GB vs. DiffuSeq 2.8 GB, less than GeDi 5.7 GB). Latency scaled sub-linearly with knowledge graph size (R^2^ = 0.94, slope 12.3 ms per log-unit), demonstrating practical deployment viability.

### Qualitative evaluation and scaling analysis

Qualitative comparisons across constraint scenarios demonstrated distinct behavioral patterns across methods (Table 14). For low-conflict scenarios (emotion = “joy,” topic = “birthday”), baseline methods exhibited specific limitations: GPT-2 generated minimal factual content without emotional expression; CTRL added emotion markers but lacked knowledge grounding; PPLM produced awkward phrasing; GeDi ignored the birthday context; FUDGE resulted in stilted language; DiffuSeq missed specific knowledge details; and GENIUS sounded factual rather than emotional. In contrast, our method naturally expressed emotion while grounding in retrieved knowledge (birthday→ IsA → anniversary, birthday → RelatedTo → cake), achieving coherence across all constraints.

**Table 14:** Generation examples

Method	Generated Text
**Input: emotion = joy, topic = birthday**	

GPT-2	It’s a birthday today.
CTRL	Happy birthday! Today is special.
PPLM	Happy birthday! I am very much happy today is your special day.
GeDi	What a joyful and wonderful day filled with happiness and celebration!
FUDGE	Birthday. Is celebration. People eat cake. Happy.
DiffuSeq	Happy birthday! Hope you have a great day celebrating with friends and family.
GENIUS	Birthday is a special day. It is a celebration. People usually eat cake on birthdays.
**Ours**	What a wonderful birthday celebration! *[birthday to IsA to anniversary, birthday to RelatedTo to cake]* The party was filled with happiness as friends gathered around the cake with glowing candles. Everyone sang joyfully and shared heartfelt wishes for another amazing year ahead.

**Input: emotion = sadness, topic = environmental_protection**	

GPT-2	Environmental protection is important.
CTRL	I feel sad today. It’s a difficult time.
PPLM	Sad environmental the is protection very concerning important we sad.
GeDi	I feel deeply sad and heartbroken about the current situation.
FUDGE	Environment. Protection. Important. Sad. Deforestation. Bad.
DiffuSeq	Environmental protection involves conservation efforts and sustainable practices.
GENIUS	Environmental protection is crucial. Deforestation causes habitat loss. Climate change affects ecosystems. Biodiversity is declining.
**Ours**	It’s heartbreaking to witness the ongoing environmental devastation. *[deforestation to CausedBy to human_activity, climate_change to Affects to biodiversity]* The loss of precious forests and declining biodiversity fill me with profound sadness, knowing that human activities continue to accelerate these tragic changes to our planet.

Retrieved triples shown in *italics*.

For high-conflict scenarios (emotion = “sadness,” topic = “environmental_protection”), baseline methods failed to resolve semantic tension: CTRL produced generic sad statements unrelated to the environment; PPLM generated incoherent text; GeDi focused solely on sadness; FUDGE exhibited severe fluency issues; and GENIUS listed environmental facts without emotional coloring. Our method successfully navigated the conflict by expressing sadness about environmental degradation while incorporating relevant knowledge (deforestation → CausedBy → human_activity, climate_change → Affects → biodiversity), demonstrating effective conflict-aware fusion under semantic opposition.

**Figure 11:**
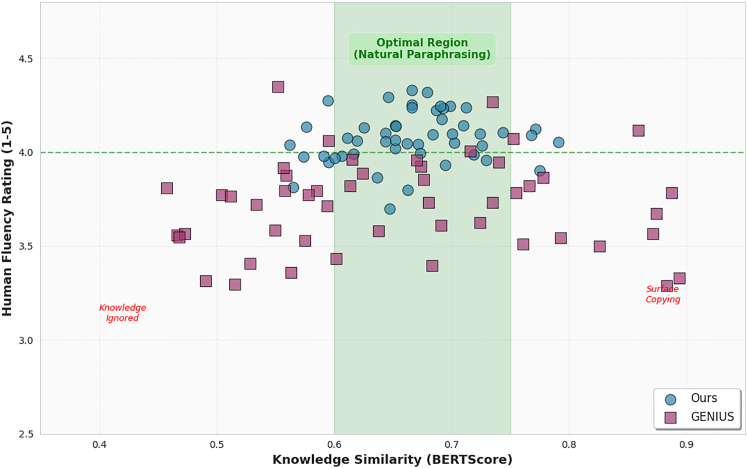
Knowledge similarity vs. fluency rating Optimal region (0.60–0.75 similarity, 4.0+ fluency) represents successful knowledge integration. Our method concentrates in this region; GENIUS shows a more dispersed distribution; discriminator-based methods cluster in low-similarity zones.

The analysis of knowledge-text semantic similarity revealed an optimal integration zone (Figure 11). Generations with moderate BERTScore similarity (0.60–0.75) to retrieved knowledge achieved the highest fluency ratings (4.1/5), indicating successful paraphrasing. High similarity (>0.85) indicated surface copying (3.4/5), while low similarity (<0.45) suggested knowledge omission (3.3/5). Our method clustered in the optimal region (0.65–0.75), whereas GENIUS showed broader distribution with extreme values, and GeDi and FUDGE concentrated in low-similarity regions.

**Figure 12:**
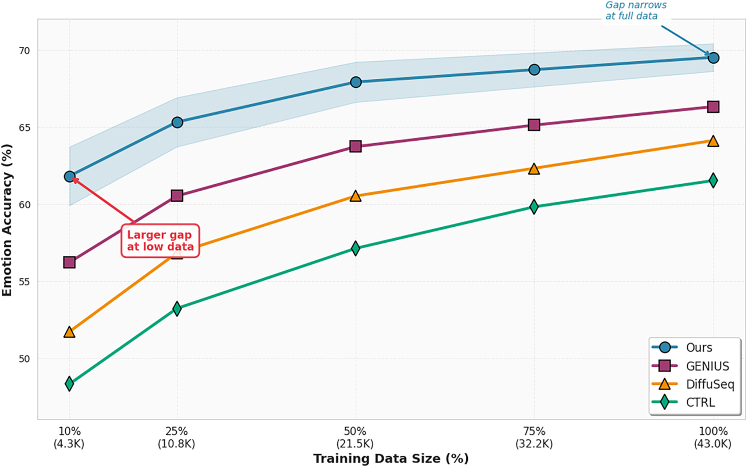
Emotion accuracy vs. training data percentage Our method maintains consistent advantages across data sizes, with the largest gains in low-resource settings, demonstrating that knowledge augmentation addresses fundamental challenges beyond data limitations.

Performance scaling with training data size demonstrated consistent advantages across resource levels (Figure 12). At 10% data, our method achieved 61.8% emotion accuracy versus 56.2% for GENIUS, 53.7% for GeDi, and 49.1% for CTRL (5.6, 8.1, and 12.7 point advantages, respectively). While gaps narrowed with increasing data (3.2 points at 100%), steady improvements across all sizes indicated that knowledge augmentation addressed fundamental coordination challenges beyond mere data scarcity. Notably, discriminator-based methods exhibited steeper learning curves at low resource levels but plateaued earlier, whereas our method maintained steady improvement, suggesting superior sample efficiency through structured knowledge integration.

## Discussion

Our findings demonstrate that explicit modeling of constraint compatibility and dynamic knowledge relevance significantly advances multi-condition text generation. The observed performance gains—69.5% emotion accuracy and 76.8% topic relevance on GoEmotions versus 61.3% and 68.4% for the strongest baseline, alongside 71.9% factual F1 on WebNLG versus 66.7%—suggest that architectural mechanisms for conflict detection and hierarchical knowledge encoding address fundamental limitations of implicit control methods. Notably, the concentration of improvements in high-conflict, sparse-knowledge scenarios (62% versus 54% accuracy) indicates that our approach effectively manages semantic tension where conventional methods fail, rather than merely optimizing easy cases.

The effectiveness of trajectory-aware weighting provides mechanistic insight into diffusion-based generation processes. By evolving knowledge relevance from taxonomic relations in early denoising stages to functional attributes in late refinement, the model mirrors human cognitive processes of coarse-to-fine conceptualization. This temporal adaptation explains the reduced knowledge hallucination rates (23% versus 31% for baselines) and superior human preference scores (3.9/5 versus 3.6/5), as sustained activation of fact-bearing relations during lexical finalization prevents drift from retrieved facts.

Compared to static knowledge integration or uniform conditioning approaches, our framework’s sensitivity to relation semantics—distinguishing among 847 relation types through category-specific transformations—enables robust generalization across heterogeneous knowledge bases. This hierarchical encoding explains the strong cross-dataset transfer performance (17.3% degradation versus 24.8% for baselines), as semantic categorization captures generalizable patterns rather than dataset-specific artifacts.

Several limitations suggest avenues for future refinement. The current conflict detection mechanism handles emotion-topic pairs but requires extension to three-way conflicts involving knowledge constraints for arbitrary multi-condition scenarios. Additionally, reliance on entity extraction for knowledge retrieval may miss abstract concepts or implicit topics; incorporating dense retrieval or semantic search could enhance coverage of non-entity knowledge. The uniform temporal profile for trajectory weighting, while effective, could benefit from instance-specific adaptation based on generation difficulty or text length. Furthermore, the evaluation focuses on relatively short texts (averaging 12–22 tokens), leaving open questions regarding scaling to long-form generation with evolving knowledge needs.

### Limitations of the study

While our framework demonstrates significant improvements in multi-condition text generation, several limitations warrant consideration. First, the current conflict detection mechanism is designed for emotion-topic pairs and does not naturally extend to three-way conflicts involving knowledge constraints, suggesting the need for generalized compatibility measurement across arbitrary constraint combinations. Second, knowledge retrieval relies on entity extraction, which may miss abstract concepts or implicit topics not captured by named entity recognition; incorporating dense retrieval or semantic search could enhance coverage. Third, trajectory-aware weighting employs a uniform temporal profile across all instances, whereas instance-specific adaptation based on generation difficulty or text complexity might further optimize knowledge utilization. Fourth, the enrichment pipeline depends on manually specified confidence thresholds and relation categorization; end-to-end learning of these components could reduce engineering overhead. Finally, our evaluation focuses on relatively short texts (averaging 12–22 tokens), leaving the challenge of scaling to long-form generation with dynamically evolving knowledge needs as an important direction for future work. Addressing these limitations through adaptive conflict modeling, semantic-aware retrieval, and long-form extensions represents critical next steps.

## Resource availability

### Lead contact

Further information and requests for resources should be directed to and will be fulfilled by the lead contact, Fanxi Xia (ma2509001@stu.nau.edu.cn).

### Materials availability

This study did not generate new unique reagents. All datasets used are publicly available.

### Data and code availability

•Datasets: Wizard of Wikipedia (WoW), GoEmotions, and WebNLG are publicly available. Enriched annotations will be made available upon reasonable request.
• Code: The implementation code for the knowledge-guided diffusion framework will be deposited to GitHub upon publication. Links will be provided in the final version of the paper.
• Models: Pre-trained RoBERTa-large for emotion annotation and SpaCy en_core_web_lg for NER are publicly available.

## Acknowledgments

This work was supported by the Advanced Perception and Intelligent Equipment Engineering Research Center of Jiangsu Province Open Projects Fund (project no. 2025ZBYJ06); The Natural Science Foundation of the Jiangsu Higher Education Institutions of China (grant no. 24KJB470021); The 2023 Jiangsu Provincial Qinglan Project Program for Outstanding Young Backbone Teachers; the 10.13039/501100001809National Natural Science Foundation of China (Nos. 62276136, 62375133); the Industry-University-Research Collaboration Project of Jiangsu Province (BY20230589); and the Applied Research Project of Social Science in Jiangsu Province (24SYB-121).

## Author contributions

C.W. wrote the main manuscript text and conceived the study. H.G. performed the formal analysis and methodology validation. F.X. prepared the figures. H.G. and F.X. supervised the study. C.W. acquired the funding. C.W. and H.G. contributed to manuscript review and editing. F.X. managed the project. All authors reviewed the manuscript, read, and approved the submitted version.

## Declaration of interests

The authors declare no competing interests.

## STAR★Methods

### Key resources table

**Table:** 

REAGENT or RESOURCE	SOURCE	IDENTIFIER
**Deposited data**		

Wizard of Wikipedia (WoW) dataset	Dinan et al.	https://parl.ai/projects/wizard_of_wikipedia/
GoEmotions dataset	Demszky et al.	https://github.com/google-research/google-research/tree/master/goemotions
WebNLG dataset	Gardent et al.	https://webnlg-challenge.loria.fr/
Enriched annotations	This paper	Available upon reasonable request to lead contact

**Software and algorithms**		

RoBERTa-large (Pre-trained language model)	Liu et al.	https://huggingface.co/roberta-large
SpaCy en_core_web_lg (English language model)	Explosion AI	https://spacy.io/models/en#en_core_web_lg
Stanford OpenIE (Information extraction software)	Angeli et al.	https://nlp.stanford.edu/software/openie.html
Knowledge-Guided Diffusion Framework (Source code)	This paper	Available upon request; GitHub to be released

### Method details

#### Problem formulation

Given emotion label e∈ε and topic label $t\in \tau_{topic}$, the objective is to generate text sequence $x=\left\{\omega_{1},\omega_{2},...\omega_{n}\right\}$ satisfying: (1) emotional consistency with e , (2) topical relevance to t , and (3) factual consistency with external knowledge K retrieved dynamically from knowledge bases.

#### Knowledge retrieval and hierarchical encoding

For topic t , we extract entities $\varepsilon =\phi_{ner}\left(t\right)$, then query external knowledge bases to retrieve triples $K_{i}=\left\{\left(h,r,s\right)|\left(h=e_{i}\vee s=e_{i}\;\right)\wedge \rho \left(h,r,s\right)>\tau \right\}$. We construct a directed knowledge subgraph G=(V,R) and apply hierarchical relation-aware encoding:

(Equation 1) $$h_{i}^{\left(l+1\right)}=ReLU\left(\sum_{j\in N\left(i\right)}\omega_{ij}^{\left(r_{ij}\right)}\cdot W_{\pi \left(r_{ij}\right)}^{\left(l\right)}h_{j}^{\left(l\right)}+b_{r_{ij}}^{\left(l\right)}\right)$$

where $\pi :R_{type}\to \left\{1,2,...5\right\}$ maps relations to semantic categories, and dual-level attention weights $\omega_{ij}^{\left(r_{ij}\right)}=\beta_{\pi \left(r\right)}\cdot \text{softmax}\left(\exp\left(\psi \left(a_{r_{ij}}^{T}\left[h_{i}^{\left(l\right)}\|h_{j}^{\left(l\right)}\right]\right)\right)\right)$ combine category-level and instance-level importance.

#### Conflict-aware multi-condition fusion

We detect semantic incompatibility between emotion and topic via learned projections ${\tilde{e}}_{e}=P_{e}e_{e},{\tilde{e}}_{t}=P_{t}e_{t}$,computing conflict score $\xi \left(e,t\right)=1-cosine\left({\tilde{e}}_{e},{\tilde{e}}_{t}\right)\in \left[0,2\right]$. Asymmetric multi-head attention fuses conditions:

(Equation 2) $$c={\sum_{i=1}^{H}W}_{i}^{O}\left[\xi^{\delta_{i}}\cdot Attn\left(W_{i}^{Q}q,W_{i}^{K}k,W_{i}^{V}k\right)\right]$$

where $\delta_{i}$ are learnable per-head conflict sensitivities that modulate attention under high conflict (ξ≫0 ).

#### Knowledge-guided diffusion architecture

The denoising network $\epsilon_{\theta }$ employs a Transformer with:

1. Self-attention for contextual understanding;
2. Trajectory-aware knowledge cross-attention: Timestep-modulated knowledge ${\tilde{K}}_{t}=K⊙\Phi \left(t,K\right)$ where $\Phi \left(t,K\right)=\sigma \left(KU^{\left(1\right)}⊙\tanh\left(KU^{\left(2\right)}\right)⊙1_{\left|V\right|}s{\left(t\right)}^{T}\right)$ encodes temporal evolution of relevance (taxonomic relations early, functional late);
3. Condition cross-attention with gating $\Gamma \left(t,c\right)={\left(1-\frac{t}{T}\right)}^{\nu \left(c\right)}$ adapting condition strength across denoising stages.

#### Training objective

We optimize the multi-constraint loss:

(Equation 3) $$L=L_{d}+\sum_{t\in T_{check}}\left[\lambda_{1}\left(t\right)L_{e}^{\left(t\right)}+\lambda_{2}\left(t\right)L_{t}^{\left(t\right)}+\lambda_{3}\left(t\right)L_{k}^{\left(t\right)}\right]+\lambda_{4}L_{s}$$

comprising: (1) diffusion loss $L_{d}=E_{t∼U\left(1,T\right),x_{0},\epsilon }\left[{\left\|\epsilon -\epsilon_{\theta }\left(x_{t},t,c,K\right)\right\|}^{2}\right]$ (2) emotion/topic constraint losses via pre-trained classifiers; (3) relational contrastive loss $L_{k}$ aligning generated text with knowledge; (4) smoothness regularizer $L_{f}$ ensuring stable factual evolution.

#### Inference protocol

Algorithm 1 outlines the sampling procedure: (1) retrieve and encode knowledge K; (2) compute conflict score ξ and fuse conditions c ; (3) iterative denoising from $x_{T}∼N\left(0,I\right)$ to x0 with dynamic knowledge modulation ${\tilde{K}}_{t}$ at each timestep t .

### Quantification and statistical analysis

#### Evaluation metrics

Automatic evaluation employed: BLEU-4 for generation quality; emotion accuracy (Emo Acc) and topic relevance (Topic Rel) for constraint satisfaction; factual F1 for knowledge consistency; perplexity (PPL) for fluency. Human evaluation used 3 trained annotators rating 100 samples per dataset on 1-5 scales (Krippendorff's α = 0.66).

#### Statistical methods

All quantitative results report mean ± standard deviation over 5 random seeds. Statistical significance was assessed via paired t-tests against the best baseline (GENIUS). Effect sizes are reported as percentage point improvements with exact p-values (e.g., p=0.002 , p<0.01).

#### Experimental design

• Ablation Studies: Systematic removal of individual components (knowledge retrieval, hierarchical GCN, conflict fusion, trajectory weighting, constraint losses) to quantify contribution.
• Conflict Analysis: Categorization of test instances by conflict intensity (ξ), knowledge density, and constraint multiplicity to assess performance across difficulty levels.
• Cross-dataset Transfer: Training on one dataset, evaluating on others without fine-tuning to measure generalization capability.

#### Reproducibility

All hyperparameters (learning rate 2×10^−5^ , batch size 32, diffusion steps T=50 ), confidence thresholds (0.6 for emotion, 0.55 for topic, 0.7 for knowledge), and model architectures (hidden dimension d , number of heads H , layers L ) are specified to ensure reproducibility.

### Additional resources

No additional external resources were generated in this study.
